# Carbonic Anhydrase 6 Gene Variation influences Oral Microbiota Composition and Caries Risk in Swedish adolescents

**DOI:** 10.1038/s41598-018-36832-z

**Published:** 2019-01-24

**Authors:** A. Esberg, S. Haworth, C. Brunius, P. Lif Holgerson, I. Johansson

**Affiliations:** 1 Department of Odontology/Section of Cariology, Umeå University, Umeå, Sweden; 2 Medical Research Council Integrative Epidemiology Unit, Department of Population Health Sciences, Bristol Medical School, University of Bristol, Bristol, United Kingdom; 3 Bristol Dental School, University of Bristol, Bristol, United Kingdom; 4 Department of Biology and Biological Engineering, Chalmers University of Technology, Gothenburg, Sweden; 5 Department of Odontology/Section of Pedodontics, Umeå University, Umeå, Sweden

## Abstract

Carbonic anhydrase VI (CA6) catalyses the reversible hydration of carbon dioxide in saliva with possible pH regulation, taste perception, and tooth formation effects. This study assessed effects of variation in the *CA6* gene on oral microbiota and specifically the acidophilic and caries-associated *Streptococcus mutans* in 17-year old Swedish adolescents (n = 154). Associations with caries status and secreted CA6 protein were also evaluated. Single Nucleotide Polymorphisms (27 SNPs in 5 haploblocks) and saliva and tooth biofilm microbiota from Illumina MiSeq 16S rDNA (V3-V4) sequencing and culturing were analysed. Haploblock 4 (rs10864376, rs3737665, rs12138897) CCC associated with low prevalence *of S. mutans* (OR (95% CI): 0.5 (0.3, 0.8)), and caries (OR 0.6 (0.3, 0.9)), whereas haploblock 4 TTG associated with high prevalence *of S. mutans* (OR: 2.7 (1.2, 5.9)) and caries (OR: 2.3 (1.2, 4.4)). The TTG-haploblock 4 (represented by rs12138897(G)) was characterized by *S. mutans*, *Scardovia wiggsiae, Treponema sp*. HOT268, *Tannerella sp*. HOT286, *Veillonell*a gp.1 compared with the CCC-haploblock 4 (represented by rs12138897(C)). Secreted CA6 in saliva was weakly linked to *CA6* gene variation. In conclusion, the results indicate that *CA6* gene polymorphisms influence *S. mutans* colonization, tooth biofilm microbiota composition and risk of dental caries in Swedish adolescents.

## Introduction

Carbonic anhydrases (CAs) are zinc enzymes that catalyse the reversible hydration of carbon dioxide in the reaction CO_2_ + H_2_O ⇔ HCO_3_^−^ + H^+^ and are important for pH homeostasis in body tissues and fluids and for removal of intracellular carbon dioxide^1^. In mammalian cells, CAs are involved in several biological processes, including HCO_3_^−^ dependent metabolic processes, secretion of electrolytes, respiration, pH regulation, bone resorption, biomineralization, and odontogenesis^2^. Today, 16 different CA isozymes have been identified in humans^1^, of which 13 have been shown to be enzymatically active. Some isozymes are expressed in most tissues, while others are tissue- or organ-specific. Five isozymes are cytosolic (I, II, III, VII and XIII), five are membrane-bound (IV, IX, XII, XIV and XV), two are present in the mitochondria (VA and VB), and one (VI, CA6) is a secreted isozyme^3,4^.

CA6 is secreted into saliva with considerable individual variation in concentration and activity^5^, and incorporated in the protein pellicle on tooth enamel and in dental biofilms^6–8^. CA6 supports neutralization of lactic and other bacteria-produced acids through conversion of saliva HCO_3_^−^ to water and carbon dioxide and pH maintenance through the subsequent phase buffering^9^. CA6 is therefore suggested to be an important enzyme in oral physiology and tooth tissues integrity, i.e., resistance to caries and dental erosion^6,10^. However, data are conflicting as both high and low concentrations and activities of CA6 have been associated with caries^4,11,12^.

Single nucleotide polymorphisms (SNPs) and multisite haploblocks in the *CA6* gene have been linked to CA6 concentration and activity in saliva and to caries status^13–17^, but findings are not consistent^18^. However, no study has confirmed the association between CA6 in saliva and *S. mutans* colonization in humans and no study has investigated *CA6* polymorphism and levels of caries associated bacteria or overall oral microbiota.

The primary aim of this study was to evaluate the effects of genetic variation in the *CA6* gene region on oral microbiota and specifically the acidophilic and caries-associated *S. mutans* in Swedish adolescents. Secondary aims were to evaluate associations between variation in the *CA6* gene region and secreted CA6 protein and caries status.

## Results

### Participant characteristics

Using next-generation sequencing (NGS), 75.3% of participants had detectable *S. mutans*, with significantly higher proportions among the caries-affected than caries-free subjects (p < 0.001; Table 1). Overall, the median caries score (DeFS) was 2.0 tooth surfaces and 67% of participants were affected by caries (DeFS ≥ 1) and 33% were caries-free (DeFS = 0) (Table 1). There were no significant differences between those with detectable *S. mutans* versus not or those who were caries-affected versus not with respect to sex, smoking, sweet snacking, BMI, and number of tooth surfaces. The proportion who reported brushing the teeth twice a day did not differ between those with *S. mutans* versus not, but it was significantly lower among caries-affected than caries-free participants (p < 0.001; Table 1).

**Table 1:** Characteristics of the 17-year-old participants all together and by *S. mutans* and caries status.

	All participants	*S. mutans* status by NGS			Caries status		
		*S. mutans* free	*S. mutans* present	p-value	Caries-free	Caries-affected	p-value
	(n = 154)	(n = 38)	(n = 116)		(n = 51)	(n = 102)	
Sex, % male	44.2	50.0	42.2	0.403	47.1	42.2	0.565
Smoker, %	2.6	0	3.5	0.244	2.0	2.9	0.600
Tooth brushing twice daily, %	83.8	89.5	81.9	0.272	98.0	76.5	**<0.001**
Sweet snack frequency/day	1.1 (0.5, 2.7)	1.2 (0.6, 3.8)	0.9 (0.4, 3.6)	0.182	1.1 (0.5, 2. 7)	1.0 (0.4, 2.6)	0.763
BMI^c^, kg/m^2^	21.6 (18.5, 26.0)	22.4 (18.2, 26.7)	21.4 (18.6, 25.9)	0.533	21.3 (18.4, 25.3)	22.0 (18.7, 26.3)	0.169
Dental status							
Number of tooth surfaces	128 (108, 128)	128 (128, 129)	128 (108, 128)	0.010	128 (118, 132)	128 (108, 128)	0.049
DeFS	2.0 (0, 17.0)	0.0 (0.0, 7.0)	3.0 (0.0, 18.4)	**<0.001**	0 (0, 0)	4.0 (1.0, 19.7)	—
*S. mutans*							
Proportion with *S. mutans* by NGS, %	75.3	—	—	—	58.8	84.3	**<0.001**
Mutans streptococci							
CFU/mL, log^10^-value	2.8 (0, 4.8)	0.0 (0.0, 3.2)	3.1 (0.0, 5.1)	**<0.001**	2.27 (0, 4.17)	2.92 (0, 5.16)	0.035
Proportion with detection by culture, %	61.4^a^	64.9	69.8		52.0	65.7	0.104
Saliva							
Protein concentration, mg/mL	0.6 (0.3, 0.9)	0.7 (0.3, 1.4)	0.5 (0.3, 0.9)	0.971	0.5 (0.3, 0.8)	0.6 (0.3, 1.0)	0.702
Flow rate, ml/min	1.5 (0.7, 2.5)	1.5 (0.2, 3.2)	1.4 (0.6, 2.5)	0.925	1.6 (0.8, 2.7)	1.4 (0.5, 2.5)	0.066
CA6 protein in saliva							
Proportion (%) of total protein in saliva	1.6 (0.1, 3.7)	1.8 (0.1, 3.8)	1.6 (0.1, 3.6)	0.430	2.0 (0.03, 4.2)	1.5 (0.08, 3.8)	0.146
Concentration, µg/mL	8.4 (0.3, 17.0)	11.5 (0.03, 35.6)	7.7 (0.3, 16.7)	0.351	9.8 (0.2, 19.4)	7.8 (0.3, 17.0)	0.203
Concentration, µg/mL high tertile, %	33.3	41.2	31.5	0.446	50.0	25.8	0.024
Secreted amounts, µg/min	11.0 (0.4, 25.4)	12.8 (1.1, 30.5)	10.6 (4.1, 25.7)	0.518	17.1 (0.2, 54.2)	8.3 (0.6, 20.2)	**0.012**
Secreted amounts, µg/min, high tertile, %	33.7	35.3	33.3	0.878	53.6	24.6	**0.007**

Caries information was missing for 1 participant who was excluded. Continuous measures are presented as medians (10, 90 percentiles) and group differences were tested with Mann Whitney U test. Differences between group numbers (presented as %) were tested with Chi^2^ or Fisher’s exact test. P-values were considered significant at FDR < 0.25 but significant differences at FDR ≤ 0.06 are also indicated in bold. CFU for colony forming units and NGS for Next Generation Sequencing of DNA extracted from saliva (n = 152) or tooth biofilm (n = 139).

### *CA6* single nucleotide polymorphic sites and gene structure

After quality control measures, 27 out of 30 Single Nucleotide Polymorphisms (SNPs) were retained for analyses. Two SNP (rs2274333 and rs17032942) were excluded due to sample call rate of 0%. An additional SNP (rs6697763) was excluded from analysis for deviation from Hardy-Weinberg equilibrium (p = 0.033), leaving 27 SNPs for further analyses.

**Figure 1:**
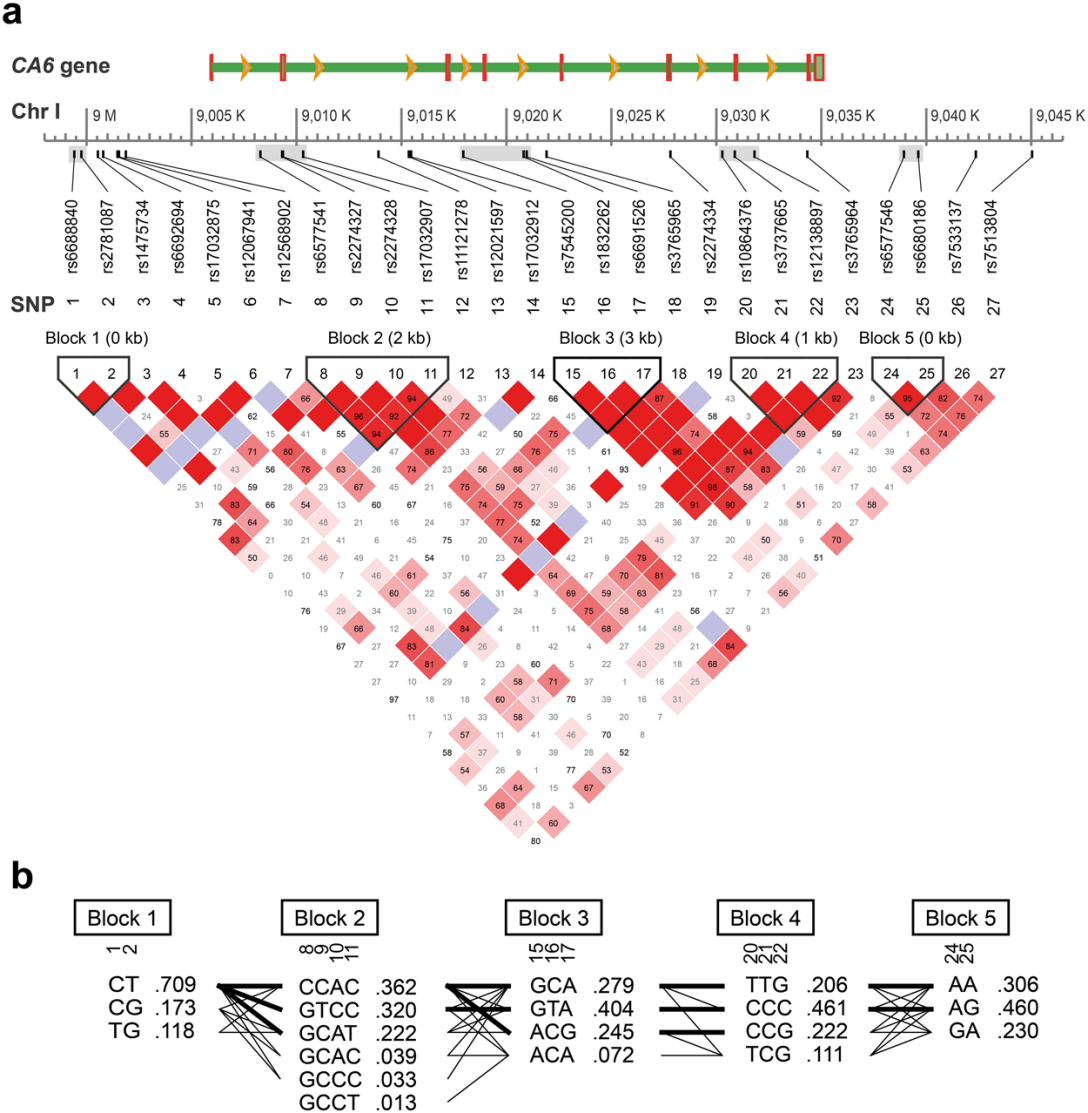
Haplotype map of *CA6* locus. (**a**) The *CA6* gene structure and distribution of selected and quality-controlled tag single nucleotide polymorphic sites (SNP). Graphics illustrates pairwise linkage disequilibrium (LD) between polymorphisms and each square display coefficient of linkage disequilibrium D′ value (%) between marker pairs. In the event of D′ = 100% where no recombination events are observed, the boxes are empty. (**b**) Haploblocks are estimated using Haploview as described in the method section. Uncommon haplotypes (observed in <1% of participants) are not shown. Genetically linked haploblocks are connected with (inherited together with >1% (thin line) or >10% (thick line) together) lines.

Evaluating potential multisite haploblocks within or around the *CA6* gene resulted in five haploblocks (Fig. 1). Haploblock 1, comprised two SNPs (rs6688840 and rs2781087) and was located upstream of the first coding exon (+6387 to +6050 bp), whereas Haploblock 5 comprised rs6577546 and rs6680186 and was located downstream of the stop codon (−3871 to −4553 bp) of the *CA6* gene. Haploblocks 2, 3 and 4 where within the *CA6* gene; haploblock 2 with four SNPs (rs6577541, rs2274327, rs2274328, and rs17032907) covered the 2477 to 4512 bp region; haploblock 3 with three SNPs (rs7545200, rs1832262, and rs6691526) covered the 12,141 to 15,160 bp region; and haploblock 4 with three SNPs (rs10864376, rs3737665, and rs12138897) covered the 24,479 to 26,010 bp region of the *CA6* gene.

### *CA6* gene variation and *S. mutans* colonization by NGS

**Figure 2:**
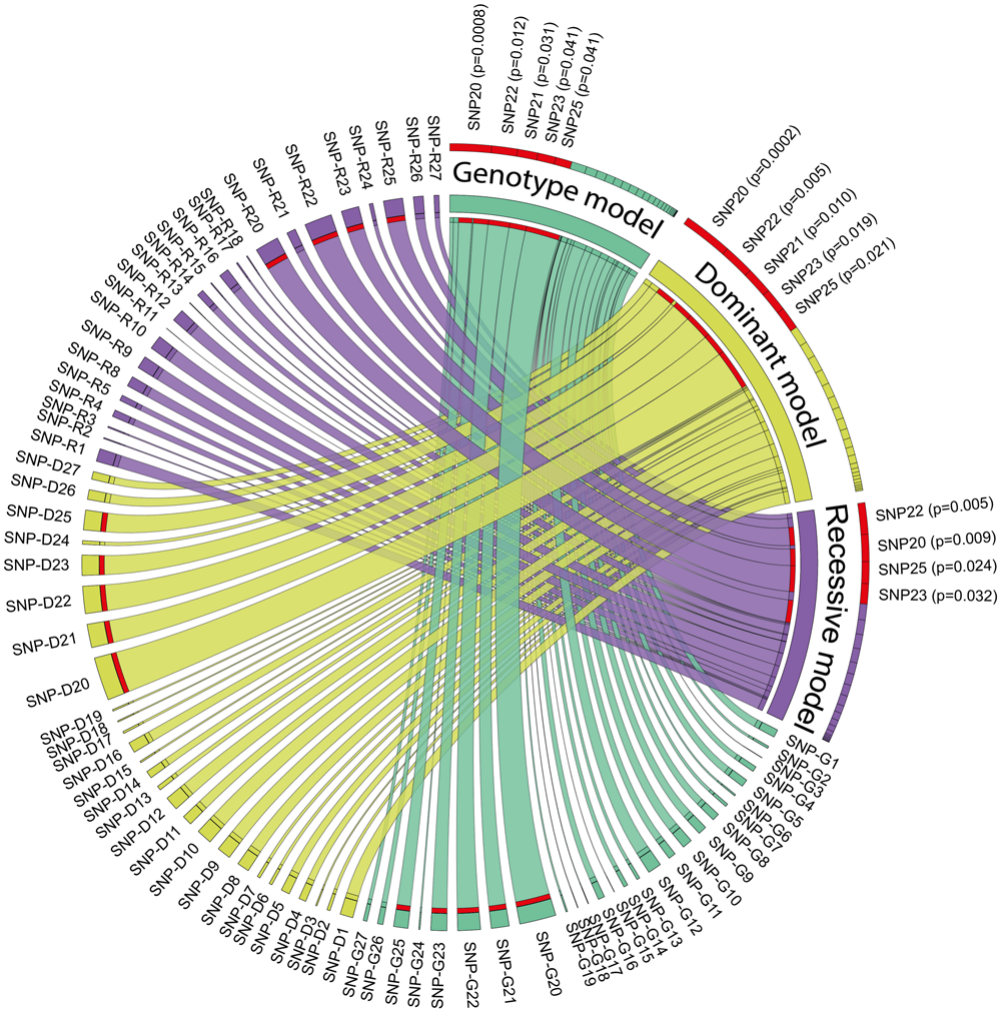
Associations between *CA6* gene variation and having detectable *S. mutans* in saliva or tooth biofilm by NGS. The association between 27 *CA6* SNPs and detectable *S. mutans* in tooth biofilm and saliva by NGS was estimated using a Chi^2^ test under 3 models; genotype model (green, SNPs marked with G), dominant model (yellow, SNPs marked with D) and recessive model (purple, SNPs marked with R). For each model, the width of the bar connecting the model name to a SNP indicates the relative–log10 p-value testing the null hypothesis of no association with detectable *S. mutans*. The red bar highlights SNPs where the null hypothesis was rejected after adjustment of p-values for multiple testing (FDR < 0.25).

Five SNPs, i.e., rs10864376 (T), rs3737665 (T), rs12138897 (G), rs3765964 (A), and rs6680186 (A) were associated with increased odds of detectable *S. mutans* in saliva or tooth biofilm by NGS (Table 2). Different genetic models (allelic-, dominant- and recessive model) yielded qualitatively similar conclusion, but with some variation in test statistics (Fig. 2).

**Table 2:** *CA6* SNP and haploblock associations with *S. mutans* detection in both saliva and tooth biofilm by NGS.

SNP					Haploblock				
SNP number	Name	Allele	Detected vs. Not detected		Haplo-block number	Allele	Frequency	Detected vs. Not detected	
			OR (95%CI)	p_chi_^2^				OR (95%CI)	p_chi_^2^
1	rs6688840	C/T	1.2 (0.5, 2.6)	0.664	Block 1	CT	0.709	1.1 (0.6, 1.9)	0.794
2	rs2781087	T/G	1.2 (0.6, 1.9)	0.794		CG	0.173	1.0 (0.5, 2.0)	0.954
3	rs1475734	T/G	1.1 (0.5, 2.4)	0.737		TG	0.118	0.8 (0.4, 1.8)	0.663
4	rs6692694	C/T	1.0 (0.5, 2.1)	0.906					
5	rs17032875	C/T	1.1 (0.6, 1.8)	0.873					
6	rs12067941	C/A	1.1 (0.5, 2.6)	0.807					
7	rs12568902	A/T	1.3 (0.5, 3.0)	0.584					
8	rs6577541	G/C	1.4 (0.8, 2.3)	0.251	Block 2	CCAC	0.362	0.7 (0.4, 1.2)	0.222
9	rs2274327	T/C	1.4 (0.8, 2.4)	0.279		GTCC	0.320	1.4 (0.8, 2.6)	0.216
10	rs2274328	C/A	1.5 (0.9, 2.6)	0.158		GCAT	0.222	0.7 (0.4, 1.3)	0.319
11	rs17032907	C/T	1.4 (0.8, 2.4)	0.230		GCAC	0.039	8.5 (0.5, 145.7)	0.047
12	rs11121278	G/C	1.4 (0.5, 3.8)	0.528		GCCC	0.033	1.3 (0.3, 6.4)	0.691
13	rs12021597*	A/G	1.1 (0.6, 1.9)	0.708		GCCT	0.013	1.0 (0.1, 9.7)	0.980
14	rs17032912	G/C	1.1 (0.5, 2.2)	0.835					
15	rs7545200	A/G	1.1 (0.6, 1.9)	0.756	Block 3	GTA	0.404	0.6 (0.4, 1.1)	0.089
16	rs1832262	C/T	1.6 (0.9, 2.6)	0.092		GCA	0.279	1.6 (0.9, 3.0)	0.124
17	rs6691526	A/G	1.1 (0.6, 2.1)	0.673		ACG	0.245	0.9 (0.5, 1.6)	0.672
18	rs3765965	T/C	1.0 (0.5, 2.0)	0.908		ACA	0.072	2.2 (0.6, 7.6)	0.206
19	rs2274334	G/T	1.2 (0.4, 4.0)	0.741					
20	**rs10864376** *	T/*C*	3.5 (1.8, 7.1)	**2.0E-4**	Block 4	CCC*	0.461	*0.5 (0.3, 0.8)*	***0.003***
21	rs3737665*	T/*C*	2.7 (1.2, 5.9)	0.012		CCG	0.222	0.8 (0.4, 1.5)	0.501
22	rs12138897*	G/*C*	2.2 (1.3, 3.7)	**0.004**		TTG*	0.206	2.7 (1.2, 5.9)	0.012
23	**rs3765964**	A/*G*	1.9 (1.1, 3.4)	**0.018**		**TCG**	0.111	3.8 (1.1, 12.8)	**0.022**
24	rs6577546	G/A	1.2 (0.6, 2.2)	0.584	Block 5	***AG***	0.460	*0.6 (0.3, 0.9)*	***0.035***
25	**rs6680186**	A/*G*	1.9 (1.1, 3.1)	0.021		AA	0.306	1.7 (0.9, 3.1)	0.079
26	rs7533137	G/*A*	1.5 (0.8, 2.9)	0.369		GA	0.230	1.3 (0.7, 2.5)	0.411
27	rs7513804	T/*C*	1.3 (0.8, 2.3)	0.283					

Alleles in bold indicate significant positive associations and in italic negative associations at FDR of < 0.25 (bold underline for differences significant at FDR ≤0.06 too). * indicates SNPs also associated with caries (see Table 4).

Further, the CCC-haploblock 4 and the AG-haploblock 5 associated with reduced odds to have *S. mutans*, whereas the GCAC-haploblock 2, and TCG and TTG haploblock 4 associated with increased odds (Table 2). The odds ratio (95% CI) to have *S. mutans* in saliva or tooth biofilm if carrying the TCG allele versus the CCC haploblock 4 was 5.0 (1.5–17.2) (p = 0.011), and if carrying the TTG allele versus the CCC haploblock 4 it was 3.3 (1.5–7.6) (p = 0.004).

### *CA6* gene variation and viable mutans streptococci in saliva

Four SNPs, rs10864376 (T), rs12138897 (G), rs6680186 (A), and rs7513804 (T), were associated with having mutans streptococci by saliva culturing (Table 3), as was the TCG-haploblock 4, whereas, the CCC-haploblock 4 and AG-haploblock 5 were associated with being *S. mutans* free (Table 3). The odds ratio (95% CI) to have mutans streptococci by culturing if carrying the TCG allele versus the CCC haploblock 4 was 4.3 (1.5–12.2) (p = 0.006). Notably, the association pattern with *CA6* gene variation was similar for having *S. mutans* by NGS and having viable *S. mutans* by culture.

**Table 3:** *CA6* SNP and haploblock associations with detection of viable mutans streptococci by saliva culturing.

SNP					Haploblock				
SNP number	Name	Allele	Detected vs. Not detected		Haplo-block number	Haplo-type	Frequency	Detected vs. Not detected	
			OR (95%CI)	p_chi_^2^				OR (95%CI)	p_chi_^2^
1	rs6688840	C/T	1.1 (0.5, 2.3)	0.709	Block 1	CT	0.709	1.1 (0.7, 1.8)	0.707
2	rs2781087	T/G	1.1 (0.7, 1.8)	0.707		CG	0.173	1.0 (0.5, 1.8)	0.895
3	rs1475734	T/G	1.9 (1.0, 3.7)	0.069		TG	0.118	0.9 (0.4, 1.8)	0.709
4	rs6692694	C/T	1.2 (0.6, 2.2)	0.613					
5	rs17032875	C/T	1.5 (0.9, 2.5)	0.107					
6	rs12067941	C/A	1.5 (0.7, 3.3)	0.272					
7	rs12568902	A/T	1.6 (0.7, 3.6)	0.213					
8	rs6577541	C/G	1.3 (0.8, 2.1)	0.275	Block 2	CCAC	0.362	1.3 (0.8, 2.1)	0.320
9	rs2274327	C/T	1.0 (0.6, 1.7)	0.886		GTCC	0.320	0.9 (0.6, 1.5)	0.736
10	rs2274328	C/A	1.1 (0.7, 1.8)	0.686		GCAT	0.222	0.7 (0.4, 1.2)	0.158
11	rs17032907	C/T	1.3 (0.8, 2.2)	0.369		GCAC	0.039	1.5 (0.4, 6.0)	0.761
12	rs11121278	C/G	1.7 (0.8, 3.7)	0.202		GCCC	0.033	1.5 (0.4, 6.0)	0.494
13	rs12021597*	A/G	1.3 (0.8, 2.2)	0.252		GCCT	0.013	1.9 (0.2, 18.7)	0.588
14	rs17032912	C/G	1.2 (0.6, 2.2)	0.655					
15	rs7545200	A/G	1.5 (0.9, 2.6)	0.093	Block 3	GTA	0.404	0.6 (0.4, 1.0)	0.076
16	rs1832262	C/T	1.6 (1.0, 2.5)	0.069		GCA	0.279	1.0 (0.6, 1.8)	0.846
17	rs6691526	G/A	1.4 (0.8, 2.4)	0.262		ACG	0.245	1.4 (0.8, 2.4)	0.262
18	rs3765965	T/C	1.1 (0.6, 2.0)	0.744		ACA	0.072	1.8 (0.7, 4.6)	0.249
19	rs2274334	T/G	2.4 (0.7, 8.8)	0.172					
20	rs10864376*	**T**/*C*	1.9 (1.1, 3.2)	**0.015**	Block 4	CCC*	0.461	0.5 (0.3, 0.8)	***0.004***
21	rs3737665*	T/*C*	1.4 (0.8, 2.4)	0.316		CCG	0.222	1.4 (0.8, 2.5)	0.499
22	rs12138897*	**G**/*C*	2.0 (1.2, 3.1)	**0.004**		TTG*	0.206	1.4 (0.8, 2.4)	0.316
23	rs3765964	A/G	1.4 (0.9, 2.2)	0.175		**TCG**	0.111	2.7 (1.1, 6.4)	0.021
24	rs6577546	G/A	1.5 (0.8, 2.6)	0.187	Block 5	***AG***	0.460	0.5 (0.3, 0.8)	***0.004***
25	**rs6680186**	**A**/*G*	2.0 (1.3, 3.2)	**0.003**		AA	0.306	1.6 (1.0, 2.7)	0.068
26	rs7533137	G/A	1.6 (0.9, 2.8)	0.099		GA	0.230	1.6 (0.9, 2.8)	0.125
27	**rs7513804**	**T**/*C*	1.9 (1.2, 3.0)	**0.008**					

Alleles in boldindicate significant positive associations and in italic negative associations at FDR < 0.25 (bold underline for significant differences at FDR ≤ 0.06). * indicates SNPs that also were associated with caries (see Table 4).

### *CA6* gene variation rs12138897 (G/C) and tooth biofilm microbiota

**Figure 3:**
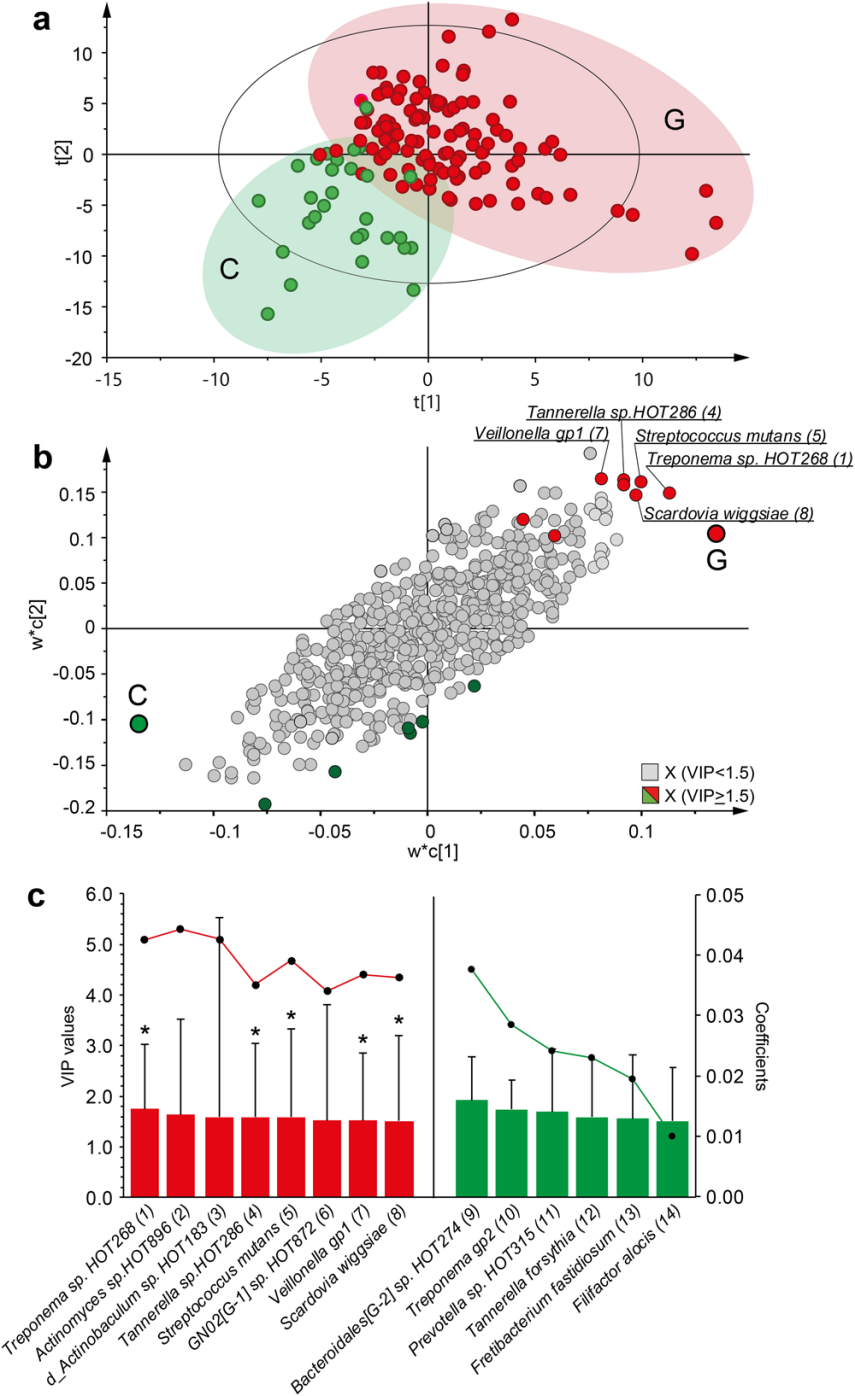
*CA6* gene variation and tooth biofilm microbiota. (**a**) PLS scatter plot illustrating separation of participants carrying either the G or C of the rs12138897 SNP based on their tooth biofilm microbiota using the dominant SNP model (G/G + G/C vs. C/C); (**b**) PLS loading scatter plot of the X and Y weights (w* and **c**) illustrating the relation between the microbiota species and the G (red) and C (green) genotype of *CA6*. Red/green dots refer to taxa with a VIP-value > 1.5, and grey dots to taxa with a VIP value < 1.5. (**c**) Correlation coefficients (mean (95% CI; shown on the left x-axis) for taxa with a VIP value > 1.5 (shown on the right x-axis) in plot B. Red bars show association with G and green bars with C genotypes of *CA6*. The dots on the lines represent median prevalence for each taxon and the stars (*) statistically significant difference between the two genotypes using univariate Chi^2^ test and p-values < 0.05.

The association of the CCC-haploblock 4 and the TCG-haploblock 4 with *S. mutans* status (as well as caries status as described in the next paragraph) led us to evaluate the association between the C and G allele of rs12138897 (C representing the CCC- and G- the TTG haploblock) and overall tooth biofilm microbiota by multivariate Partial Least Squares (PLS) analysis of NGS determined taxa. PLS modelling (crossvalidated prediction Q^2^ = 24%) displayed separation of participants carrying the G versus C allele based on their tooth biofilm microbiota (Fig. 3a). The separation was explained by strong associations (Variable Importance in Projection (VIP) > 1.5) with the G allele for eight species, i.e., higher detection rate of *Treponema sp*. HOT268, *Actinomyces sp*. HOT896, *Tannerella sp*. HOT286, *S. mutans*, GN02[G-1] *sp*. HOT872, *Scardovia wiggsiae*, and species detected by Veillonella genus probe1 (Fig. 3b). The C allele associated with higher detection rate of *Bacteroidales* [G-2] *sp*. HOT274, *Prevotella* sp. HOT315, *Tannerella forsythia*, *Fretibacterium fastidiosum*, *Filifactor alocis*, and species detected by *Treponema* genus probe 2 (Fig. 3c). Five of the eight species associating with the G allele, i.e., *Treponema* sp. HOT268, *Tannerella* sp. HOT286, *S. mutans*, *Veillonell*a gp.1 and *S. wiggsiae* were also significantly different between the G and C genotype (p_chi2_ < 0.05) in univariate analysis.

### *CA6* gene variation and caries status

Following that *CA6* gene variation was associated with colonization of caries associated bacteria, such as *S. mutans* and *S. wiggsiae*, the association between the 27 SNPs and caries status (yes or no) was tested in an allelic association model. The rs12021597 (A), rs10864376 (T), rs3737665 (T), and rs12138897 (G) associated positively with having caries (Table 4). The strongest effects on having caries was observed for the rs10864376 (T) allele with an odds ratio (95% CI) of 2.1 (1.2, 3.7) (p = 0.009).

**Table 4:** *CA6* SNP and haploblock associations with being caries-affected or caries-free.

SNP					Haploblock				
SNP number	Name	Allele	Caries-affected vs. Caries-free		Haplo-block number	Allele	Frequency	Caries-affected vs. Caries-free	
			OR (95%CI)	p_chi_^2^				OR (95%CI)	p_chi_^2^
1	rs6688840	T/C	1.1 (0.5, 2.3)	0.895	Block 1	CT	0.717	0.9 (0.5, 1.6)	0.748
2	rs2781087	G/T	1.1 (0.6, 1.9)	0.749		CG	0.167	1.1 (0.6, 2.2)	0.784
3	rs1475734	G/T	1.0 (0.5, 2.1)	0.955		TG	0.116	1.1 (0.5, 2.3)	0.894
4	rs6692694	C/T	1.1 (0.6, 2.1)	0.783					
5	rs17032875	C/T	1.1 (0.6, 1.8)	0.744					
6	rs12067941	C/A	1.2 (0.5, 2.6)	0.663					
7	rs12568902	A/T	1.0 (0.4, 2.5)	0.955					
8	rs6577541	G/C	1.1 (0.6, 1.8)	0.821	Block 2	CCAC	0.348	1.0 (0.6, 1.7)	0.948
9	rs2274327	C/T	1.2 (0.7, 2.0)	0.514		GTCC	0.329	0.8 (0.5, 1.4)	0.449
10	rs2274328	C/A	1.0 (0.5, 1.7)	0.982		GCAT	0.220	0.9 (0.5, 1.7)	0.828
11	rs17032907	C/T	1.1 (0.6, 1.9)	0.815		GCAC	0.043	1.7 (0.5, 6.8)	0.479
12	rs11121278	G/C	1.4 (0.6, 3.5)	0.468		GCCC	0.036	5.4 (0.7, 43.5)	0.065
13	**rs12021597**	**A**/*G*	1.9 (1.1, 3.4)	**0.023**		GCCT	0.011	1.3 (0.1, 14.4)	0.854
14	rs17032912	G/C	1.5 (0.8, 3.0)	0.214					
15	rs7545200	G/A	1.0 (0.6, 1.8)	0.930	Block 3	GTA	0.401	0.6 (0.4, 1.1)	0.063
16	rs1832262	C/T	1.7 (1.0, 2.8)	0.057		**GCA**	0.296	1.9 (1.1, 3.3)	**0.037**
17	rs6691526	A/G	1.2 (0.6, 2.1)	0.637		ACG	0.240	0.9 (0.5, 1.6)	0.636
18	rs3765965	T/C	1.2 (0.6, 2.3)	0.538		ACA	0.062	1.4 (0.5, 4.3)	0.508
19	rs2274334	G/T	1.1 (0.4, 3.6)	0.850					
20	**rs10864376**	**T**/*C*	2.1 (1.2, 3.7)	**0.009**	Block 4	***CCC***	0.461	0.6 (0.3, 0.9)	***0.028***
21	**rs3737665**	**T**/*C*	2.3 (1.2, 4.4)	**0.014**		CCG	0.217	0.9 (0.5, 1.7)	0.776
22	**rs12138897**	**G**/*C*	1.8 (1.1, 2.9)	**0.028**		**TTG**	0.217	2.3 (1.2, 4.4)	**0.014**
23	rs3765964	A/G	1.5 (0.9, 2.4)	0.160		TCG	0.105	1.3 (0.6, 3.1)	0.518
24	rs6577546	A/G	0.9 (0.5, 1.7)	0.851	Block 5	AG	0.475	0.8 (0.5, 1.3)	0.314
25	rs6680186	A/G	1.4 (0.8, 2.2)	0.242		AA	0.301	1.3 (0.8, 2.4)	0.319
26	rs7533137	G/A	1.1 (0.6, 2.1)	0.697		GA	0.219	1.1 (0.6, 2.0)	0.754
27	rs7513804	T/C	1.2 (0.7, 1.9)	0.573					

Alleles in bold indicate significant positive associations and in italic negative associations at FDR of < 0.25 (bold underline for significant differences at FDR ≤ 0.06).

Of the 5 haploblocks, two (haploblock 3 and 4) were associated with caries status (Table 4). The GCA-haploblock 3 and TTG-haploblock 4 associated with increased odds to have caries, whereas CCC-haploblock 4 associated with decreased odds of having caries. Comparing the TTG-haploblock 4 with the CCC-haploblock 4 showed an odds ratio of 2.6 (1.3, 5.2) (p = 0.008) covering 66.7% of the participants.

### *CA6* gene variation and saliva CA6 protein levels

Saliva concentration of CA6 was measured by ELISA in the first 90 sampled participants (for further information see material and methods). The median (10%, 90% percentile) concentration of CA6 in saliva was 8.4 (0.3, 17.0) µg/mL and secreted CA6 11.0 (0.4, 25.4) µg/min (Table 1). High concentration tended to associate with rs10864376 (T) (p = 0.031) and rs3737665 (T) (p = 0.031), as well as the GCA-haploblock 3 (p = 0.030) and TTG-haploblock 4 (p = 0.051) but none was significant at FDR < 0.25 (Table S3).

CA6 in saliva (concentration or secreted amounts) was not associated with having *S. mutans* or not, but secreted amounts (µg/min) was significantly higher in caries-free than caries-affected (p = 0.012) (Table 1). In accordance, twice as many caries-free, compared to caries-affected, participants were identified in the highest tertile based on the distribution of secreted CA6 amounts (p_between groups_ = 0.007) with an OR to be caries-free of 3.5 (1.4, 9.1).

### Haploblock 4 summary

A summary of characteristics for the various allele variants in haploblock 4 is presented in Table 5 together with comparisons between the allele variants groups as well as trends among the allele variants. There were no significant differences between the alleles with respect to sex, BMI, smoking, frequency of sweet snacking, or number of tooth surfaces. The proportions with *S. mutans* by NGS and culturing, respectively, differed significantly between the allele variant groups (p < 0.001) as did the trend from the TCG to CCC variant (p < 0.001). The association with caries was less strong, but the proportions caries-affected participants tended to differ between the allele variant groups (p = 0.054) with a significant trend (p = 0.021), whereas caries prevalence (DeFS) did not differ between the groups though the trend did (p = 0.018).

**Table 5:** Characteristics of the 17-year-old participants in Haploblock 4.

	Haploblock 4				p value	
	TCG	TTG	CCG	CCC	Group	Trend
Frequency, %	11.1	20.6	22.2	46.1	—	—
Sex, % male	77.8	58.9	55.6	54.6	0.166	0.067
Smoker, %	0.0	3.6	1.8	2.5	0.776	0.749
BMI, kg/m^2^	20.8 (17.5, 26.2)	21.1 (18.9, 25.8)	21.0 (18.2, 24.3)	21.8 (18.5, 25.2)	0.240	0.149
Sweet snack frequency/day	0.9 (0.3, 2.1)	0.7 (0.2, 1.3)	0.9 (0.3, 2.1)	0.7 (0.3, 2.2)	0.126	0.176
Number of tooth surfaces	128 (108, 128)	128 (108, 130)	128 (108, 128)	128 (113, 128)	0.364	0.136
*S. mutans*						
Proportion with detection by NGS, %	92.6	87.5	71.4	63.9	**<0.001**	**<0.001**
Mutans streptococci						
CFU/mL, log^10^ value	3.0 (0, 5.2)	2.8 (0, 4.6)	2.9 (0, 5.1)	0.7 (0, 4.4)	0.021	0.014
Proportion with detection by culture, %	81.5	64.3	69.6	50.4	**0.007**	**0.002**
Caries status						
DeFS	1.0 (0.0, 14.0)	2.0 (0.0, 13.9)	1.5 (0.0, 12.0)	1.0 (0.0, 10.0)	0.101	0.021
Proportion caries-affected, %	66.7	75.0	59.3	53.8	0.054	0.018

Continuous measures are presented as medians (10, 90 percentiles) and group differences were tested with Mann Whitney U test. Differences between group numbers (presented as %) were tested with Chi^2^ or Fisher’s exact test. P-values were considered significant at FDR < 0.25 but significant differences at FDR ≤0.06 are also indicated in bold. CFU for colony forming units and NGS for Next Generation Sequencing of DNA extracted from saliva (n = 152) or tooth biofilm (n = 139). All analysis included participants who reported brushing their teeth twice daily.

## Discussion

This study tested the hypothesis that variation in the *CA6* gene is relevant to dental health and found that *CA6* gene polymorphisms and haploblocks of *CA6* were associated with *S. mutans* colonization, overall microbiota composition and dental caries in Swedish adolescents. Secreted CA6 in saliva tended to be linked to *CA6* gene variation. Thereby, it is the first study to demonstrate effects of *CA6* gene polymorphisms on the oral bacterial communities in a population-based setting.

Collectively, the present findings support a role of *CA6* polymorphisms in oral health, i.e., balancing less, against more, aciduric species, e.g., cariogenic *S. mutans* and *S. wiggsiae*, and de- and remineralization in the caries process. The results are in line with previous studies reporting a role of CA6 in caries development in children^13–17^. Though several of the physiological effects of CA6 are relevant for both oral microbiota ecology and the caries process, the detailed mechanisms for the effects remain to be elucidated. The underlying mechanisms for oral health of CA6 and CA6 gene variations may, alone or in concert, include tooth formation, local pH regulation, bacteria adhesion and metabolism and taste induced food preferences.

A tooth formation mechanism appears plausible because CA6 is expressed in the bud, cup, and bell stage of early tooth development, and by pre- and early differentiated odontoblasts and dental papilla mesenchyme cells, but most avidly in late tooth development with formation and maturation of the enamel^2^. Differences in enamel quality affects the initial tooth covering protein pellicle, and consequently the profile of attachment epitopes for bacteria^19^ and possibly oral microbiota composition. Differences in enamel quality also affects resistance of tooth tissue to destruction in an acidic environment.

Given the importance of pH homeostasis for the oral environment, local pH regulation by CA6 may be a contributory mechanism. Several of the most abundant bacteria in the mouth are glycolytic with avid acid formation, leading to pH fall and enrichment of aciduric, cariogenic species (dysbiosis)^20^. The CA6 protein is suggested to regulate pH in secreted fluids^1^, and saliva buffer capacity was reported to differ by *CA6* gene variation at rs2274327^16^, and CA6 protein activity in some^12^, but not other^5^, studies. This aspect could not be tested in the present study as neither saliva buffer capacity nor pH were analyzed, but some gene variants were associated with presence of aciduric and acidogenic species, such as *S. mutans* and *S. wiggisae*, which are promoted at low pH. Since reported intake of sweets did not differ by CA6 gene variation this may reflect a less well functioning pH regulation by some CA6 gene variants. Impaired pH regulation may also increase or decrease the risk of tooth de- and remineralisation.

Other important factors in oral biofilm ecology include access to attachment sites on host- and partner bacteria structures, and exposure to oxygen and nutrients. CA6 incorporated in pellicle and tooth biofilm may influence bacterial ecology and metabolism through effects unrelated to it’s enzymatic activity. Such effects may be two-fold; i.e., by proteolytic release of bioactive peptides or by providing a bacteria binding site. Here, the TTG and CCC associated SNPs were linked with mixed panels of oral bacterial such as *S. mutans* and *S. wiggsiae*, but also *Treponema sp*. HOT268, and *Tannerella sp*. HOT286 which thrive at a neutral to slightly basic pH. However, these suggested mechanisms are theoretical and need to be evaluated in future studies.

Finally, *CA6* gene variation has been suggested to link to bitter taste and smell perception^21–27^. To date, reported associations with food selection are inconsistent and the present study did not find any effect of *CA6* polymorphisms on sweet snack intake. This may reflect a “true” lack of association or the inherent error in measuring sugar intake in general and especially in the dental office. This aspect as well as the reported association between bitter taste receptor stimulation and innate immunity function^28^ should be addressed by a study design that is less prone to bias, such as using a biomarker or genetic marker reflecting sweet intake.

Compared to previous investigations, the present study included detailed genotyping of a relatively large number of SNPs within and near *CA6*, allowing fine-mapping of both single genetic variants and recombination blocks. The phenotypes were also detailed, including a range of salivary, microbiome, and host characteristics which may affect oral microbiota ecology and the caries process directly or indirectly. Additional strengths were that (i) all participants were of the same age (17 years old), (ii) participants were recruited in connection with their regular dental survey which reduces the risk for selection bias, (iii) participants permanent teeth have been exposed to the oral environment for years allowing ecology stabilization and potential development caries, and (iv) have minimal confounding by diseases or medications. Further, the study design, i.e., consecutive inclusion of participants as they attended their regular visit for a dental examination, and that virtually all accepted to participate, limited the risk of a selection bias.

There are some limitations in the study that should be acknowledged. The relative low number of participants, including that only a subset was available for assessing CA6 amounts, limits the possibility of comparisons in subgroups. Further, identification of microorganisms by NGS is associated with potential errors at various steps, such as the PCR amplification, sequencing per se, and bioinformatic accuracy. It should also be noted that in contrast to culturing, DNA based identification by NGS identifies dead and alive bacteria, and that saliva detection, in contrast to tooth biofilm samples, reflects bacteria from epithelial and tooth surfaces. These differences contributed to the variation in *S. mutans* detection frequency between the different identification strategies. To reduce the impact of the sampling source, being *S. mutans* positive was based on presence in either saliva or tooth biofilm by NGS. In addition, self-reported information on lifestyle habits, here tooth brushing, tobacco use and intake of sweet snacks, is prone to bias, such as the well-known under-reporting of diet intake^29^.

CA6 was originally found in the salivary glands, but has now been found to be expressed by the lacrimal, tracheobronchial, nasal, and mammary glands (with especially high content in colostrum^30^, and in epithelial linings of the oesophagus, stomach, and large intestine. This has led to a wider search of its functions. Thereby, CA6 has been linked to acinic cell carcinoma^31^, colorectal cancer^27,32–34^, and Sjögrens syndrome^35–37^. Based on the findings from the current study it may be hypothesized that CA6 and *CA6* gene variations contribute to the microbial environment in both the nasopharynx and gastro-intestinal tracts.

In conclusion, the results from the present study support that *CA6* gene polymorphisms rs10864376 (T), rs3737665 (T), rs12138897 (G) and haploblock TTG of *CA6* are associated with *S. mutans* colonization, overall microbiota composition and dental caries in Swedish adolescents. Further studies need to elucidate the mechanisms.

## Material and Methods

### Participants

In this study 154 17-year-old adolescents were recruited from three public dental health care clinics in the city of Umeå, Sweden^38^. Adolescents who had other chronic medical conditions, used medication regularly, required antibiotic treatment during the latest six months, who did not consent, or were unable to communicate in Swedish or English were excluded. All participants had answered a questionnaire with information on general health status, medication use, oral hygiene, dietary habits, and tobacco use, and samples of whole simulated saliva and supragingival biofilm were collected and stored at −80 °C until use.

### Ethical approval

The study was approved by the Regional Ethical Review Board in Umeå, Sweden (Dnr 2012-111-31 M, Dnr 2015-389-32 M and Dnr 2017-450-31) and abided by the Declaration of Helsinki and by the European General Data Protection Regulation (GDPR). Data collections and experiments were performed in accordance with relevant guidelines and regulations, including that all adolescents and their caregivers provided informed consent and data collection and handling followed the Helsinki declaration and the Swedish Law on personal data act (PuL).

### *CA6* genotyping

Single nucleotide polymorphisms in the region of the CA6 gene were selected and genotyped on DNA isolated from whole stimulated saliva. The criteria for SNP selection were a) minor allele frequency (MAF) ≥ 5% in the CEU population b) not in complete linkage disequilibrium (r^2^ ≤ 0.8) and c) in the 10,000 bp up-stream to 10,000 bp down-stream region of the *CA6* gene. Using the NIH snptag resource (https://snpinfo.niehs.nih.gov/snpinfo/snptag.html) 30 SNPs met these criteria. Genotyping was performed using a multiplexed primer extension chemistry of the iPLEX assay with detection of the incorporated allele by mass spectrometry using a MassARRAY analyzer (Agena Bioscience, Hamburg, Germany). Raw data from the mass reader was converted to genotype data using the Typer software (Agena Bioscience)^39–41^. Two SNP markers had sample call rate 0%, rs2274333 and rs17032942, the reasons for the failure was low allele signals resulting in inaccurate genotype calls. An additional SNP (rs6697763) was excluded from analysis for deviation from Hardy-Weinberg equilibrium (p = 0.033), leaving 27 SNPs with valid genotype data, where the average call rate per sample was 99.4% and overall call rate was 99.8% (Table S1). Genotyping data are uploaded and available at figshare 10.6084/m9.figshare.6948020.

### Sampling for microbiota analyses and CA6 determination

Whole simulated saliva was collected for 3 min into ice-chilled sterile test tubes while participants chewed on a 1 g piece of paraffin wax. Supragingival biofilm was collected from all accessible tooth surfaces using sterile wooden toothpicks and pooled by participant into 100 µL of TE buffer (10 mM Tris, 1 mM ethylenediaminetetraacetic acid [EDTA], pH 7.6). All study participants refrained from oral hygiene on the morning of their visit to the dental clinic. All samples, except those for culturing, were stored at −80 °C until used.

### Oral microbiota genotyping

Genomic DNA was extracted from supragingival biofilm samples, saliva and from bacteria mock communities with the GenElute Bacterial Genomic DNA Kit (Sigma-Aldrich, Stockholm, Sweden) as previously described^38^. Briefly, samples were collected and lysed in buffer with lysozyme and mutanolysin (Sigma-Aldrich), treated with RNase and proteinase K, purified and washed. DNA quantity and quality were measured using NanoDrop 1000 Spectrophotometer (Thermo Fisher Scientific, Uppsala, Sweden). Multiplex 16S rDNA amplicon sequencing was performed with the Illumina MiSeq platform (http://www.illumina.com). 16S rDNA amplicon sequencing was conducted at the Forsyth Research Institute with the HOM*INGS* protocol^42^. Briefly, the V3-V4 hypervariable regions of the 16S rRNA gene were polymerase chain reaction (PCR) amplified with the 341 F (ACGGGAGGCAGCAG) forward primer and the 806 R (GGACTACHVGGGTWTCTAAT) reverse primer. Pair-end reads were fused using FLASH, and barcodes, primers, and ambiguous and chimeric sequences were removed within QIIME. Taxa were identified with the ProbeSeq customized BLAST program, which targeted recognition of 538 species (using 638 species-level probes) and 129 groups of closely-related species (using 129 genus-level probes)^43^. Taxa targeted by the genus probes (gp) are described in Table S2. Based on sequencing results for the mock communities, taxa that were represented by at least 100 sequences with >98.5% similarity were included in further analyses.

### Caries scoring and lifestyle information

Visual and radiographic dental examinations were performed as described previously^38^. The numbers of tooth surfaces with caries in the enamel or into the dentine, or a filling, or were missing were recorded. The total numbers of decayed and filled tooth surfaces (DeFS) were calculated. Fissure sealants were not recorded. Missing surfaces were not considered to represent dental caries because tooth loss occurred for orthodontic reasons, tooth formation defects or aplasia in the study group.

### Determination of CA6 concentration in saliva

For the first 90 participants’ saliva, samples were collected and stored at −80 °C until used for CA6 concentration measurements. CA6 protein concentration in saliva was measured by ELISA (CA6 ELISA Kit, OKCD01736, Nordic Biosite, Täby, Sweden) following the manufacturer’s instructions. Briefly, 100 µL saliva, diluted 1:5 was used for analysis, CA6 protein was captured by a target specific antibody, then detected by a biotinylated secondary antibody, followed by an avidin-horseradish peroxidase (HRP) conjugate and tetramethylbenzidine (TMB) for colour development. The colour development reaction was terminated by sulfuric acid addition and the optical density (OD) measured at 450 nm using a MultiscanGO spectrophotometry (Thermo Scientific, Abninolab, Upplands-Väsby, Sweden). A standard curve using purified CA6 protein ranging from 0–1,000 pg/mL was run.

### Determination of total saliva protein concentration

Total saliva protein concentration was determined using Pierce Coomassie (Bradford) protein assay kit (ThermoFisher). Briefly, 5 µl diluted (1:1) saliva, was mixed with 250 µL protein dye solution and incubated for 30 min at room temperature. The optical density of each sample at 595 nm wavelength was assessed using a MultiscanGO spectrophotometry (Thermo Scientific) and compared to the optical density of a bovine serum albumin standard curve from known antigen concentrations to determine the sample concentration.

### Data handling and statistical inference

NGS sequencing of saliva was performed in 139 tooth biofilm samples and 154 saliva samples where 2 samples failed. Caries information was missing for one participant who was excluded. All analyses with caries as outcome were restricted to the 129 subjects who reported brushing their teeth twice a day.

SPSS software version 24.0 was used for descriptive statistics, including medians (10%, 90% percentiles), frequencies (n), proportions (%) and odds ratios (OR) with 95% confidence intervals (CI). Group comparisons of continuous variables were done using Mann-Whitney U test and for categorical variables Chi^2^ or Fisher’s exact test. All tests were two-tailed. Correction for multiple comparison was done by the Benjamini Hochberg false discovery rate (FDR). P-values were considered significant at FDR < 0.25 and ≤0.06. The higher FDR was applied to avoid missing potential *CA6* SNPs of interest as described earlier^44,45^.

Haploview software (version 4.2) was used to evaluate characteristics of SNPs (observed heterozygosity, predicted heterozygosity, Hardy-Weinberg equilibrium, minor allele frequency, pairwise linkage disequilibrium), as well as evaluation of potential haploblocks using the algorithm described by Gabriel *et al*.^46^. Haploblocks with frequencies <1% were not included in the analyses. Association between genetic variation (SNPs and haploblocks) and phenotypes was evaluated using Haploview and Chi2 tests. The tests were two-tailed tests and corrected for multiple comparison by FDR as described above. Phenotype penetrance was assessed in common allele, recessive and dominant models^47^.

Clustering of participants by presence (0/1) of bacterial taxa in saliva and tooth biofilm, respectively, was modelled using partial least square (PLS) regression. Saliva taxa did not yield a significant model and therefore PLS analyses employed tooth biofilm taxa. Variables with a Variable Importance in the Projection (VIP) > 1.5 were considered influential. SIMCA P+ version 14.1 (Umetrics AB) was used for these analyses.

## Electronic supplementary material

Carbonic Anhydrase 6 Gene Variation influences Oral Microbiota Composition and Caries Risk in Swedish adolescents

Table S2

## Data Availability

NGS sequence data can be found at 10.6084/m9.figshare.5794989. Genotyping *CA6* datasets generated and analysed during the current study are available from the corresponding author on reasonable request and with appropriate ethical approvals.
