# Supplementary material for: Carbonic Anhydrase 6 Gene Variation influences Oral Microbiota Composition and Caries Risk in Swedish adolescents
Source: Sci Rep. 2019 Jan 24;9:452. doi: 10.1038/s41598-018-36832-z (PMC6345836; doi:10.1038/s41598-018-36832-z)
Supplement: Supplementary file 1 — Carbonic Anhydrase 6 Gene Variation influences Oral Microbiota Composition and Caries Risk in Swedish adolescents [file 41598_2018_36832_MOESM1_ESM.pdf]

Supplemental information

Carbonic Anhydrase 6 Gene Variation influences Oral Microbiota Composition and Caries Risk in Swedish adolescents

Esberg A[a], Haworth S[b,c], Brunius C[d], Lif Holgerson P[e], and Johansson I[a]

[a] Department of Odontology/Section of Cariology, Umeå University, Umeå, Sweden

[b] Medical Research Council Integrative Epidemiology Unit, Department of Population Health Sciences, Bristol Medical School, University of Bristol, Bristol, United Kingdom

[c] Bristol Dental School, University of Bristol, Bristol, United Kingdom

[d] Department of Biology and Biological Engineering, Chalmers University of Technology, Gothenburg, Sweden

[e] Department of Odontology/Section of Pedodontics, Umeå University, Umeå, Sweden

Table S1. Characteristics of genotyped *CA6* single nnucleotide polymorphisms

#	Name	Position	ObsHET[a]	PredHET[b]	HWpval[c]	%Geno[d]	MAF[e]	Alleles[f]
1	rs6688840	8999506	0.222	0.208	0.710	100.0	0.118	C:T
2	rs2781087	8999843	0.386	0.413	0.509	100.0	0.291	T:G
3	rs1475734	9000628	0.248	0.265	0.596	100.0	0.157	G:T
4	rs6692694	9000882	0.281	0.278	1.000	100.0	0.167	C:T
5	rs17032875	9001562	0.477	0.455	0.700	100.0	0.350	T:C
6	rs12067941	9001631	0.183	0.177	1.000	100.0	0.098	C:A
7	rs12568902	9001961	0.184	0.167	0.508	99.3	0.092	A:T
8	rs6577541	9008370	0.471	0.464	1.000	100.0	0.366	G:C
9	rs2274327	9009406	0.444	0.44	1.000	100.0	0.327	C:T
10	rs2274328	9009444	0.466	0.473	0.958	95.4	0.384	A:C
11	rs17032907	9010405	0.359	0.37	0.845	100.0	0.245	C:T
12	rs11121278	9013992	0.177	0.173	1.000	92.2	0.096	C:G
13	rs12021597	9015436	0.444	0.444	1.000	100.0	0.333	G:A
14	rs17032912	9015550	0.275	0.273	1.000	100.0	0.163	G:C
15	rs7545200	9018034	0.412	0.433	0.639	100.0	0.317	G:A
16	rs1832262	9020905	0.507	0.482	0.671	99.3	0.405	C:T
17	rs6691526	9021053	0.399	0.37	0.490	100.0	0.245	A:G
18	rs3765965	9021998	0.320	0.311	0.981	100.0	0.193	T:C
19	rs2274334	9027899	0.092	0.087	1.000	100.0	0.046	G:T
20	rs10864376	9030372	0.412	0.433	0.639	100.0	0.317	C:T
21	rs3737665	9030964	0.359	0.327	0.345	100.0	0.206	C:T
22	rs12138897	9031903	0.464	0.497	0.485	100.0	0.461	G:C
23	rs3765964	9034421	0.523	0.487	0.475	100.0	0.418	G:A
24	rs6697763[g]	9037273	0.405	0.495	0.033	100.0	0.451	C:T
25	rs6577546	9039022	0.375	0.358	0.765	99.3	0.234	A:G
26	rs6680186	9039704	0.458	0.497	0.383	100.0	0.464	A:G
27	rs7533137	9042447	0.392	0.36	0.400	100.0	0.235	A:G
28	rs7513804	9045124	0.490	0.472	0.799	100.0	0.382	T:C

[a] ObsHET is the marker's observed heterozygosity

[b] PredHET is the marker's predicted heterozygosity (i.e. 2*MAF*(1-MAF))

[c] HWpval is the Hardy-Weinberg equilibrium p value, which is the probability that its deviation from H-W equilibrium could be explained by chance.

[d] %Geno is the percentage of non-missing genotypes for this marker

[e] MAF is the minor allele frequency (using founders only) for this marker

[f] Alleles are the major and minor alleles for this marker

[g] Excluded from further analysis based on H-W equilibrium

Table S2. Taxa targeted by the genus probes (Separate PDF file)

Table S3. SNPs and Haploblock associating with CA6 saliva amount in 154 subjects. Gray boxes indicate haploblocks 1-5. Alleles in red indicate significant positive associations and in green negative associations at FDR of <0.25 (bold for significant differences at FDR ≤0.06).

SNP					Haploblock				
		High vs. Low CA6 amount					High vs. Low CA6 amount		
No	Name	Allele	OR (95%CI)	p_{CHI2}	Block	Haplotype	Frequency	OR (95%CI)	p_{CHI2}
1	rs6688840	T/C	1.1 (0.4-2.8)	0.897	Block 1	CT	0.672	0.9 (0.5-1.8)	0.789
2	rs2781087	G/T	1.1 (0.6-2.1)	0.789		CG	0.217	1.1 (0.5-2.3)	0.837
3	rs1475734	T/G	1.7 (0.7-3.9)	0.200		TG	0.111	1.1 (0.4-2.8)	0.897
4	rs6692694	C/T	2.0 (0.8-5.0)	0.118					
5	rs17032875	C/T	1.1 (0.5-2.0)	0.884					
6	rs12067941	C/A	1.6 (0.6-4.6)	0.382					
7	rs12568902	T/A	2.0 (0.8-4.9)	0.151					
8	rs6577541	C/G	1.5 (0.8-2.8)	0.218	Block 2	CCAC	0.361	1.4 (0.7-2.6)	0.309
9	rs2274327	C/T	1.5 (0.8-3.1)	0.225		GTCC	0.305	0.6 (0.3-1.2)	0.147
10	rs2274328	C/A	1.6 (0.8-3.2)	0.165		GCAT	0.216	1.3 (0.5-2.4)	0.730
11	rs17032907	T/C	1.3 (0.7-2.7)	0.425		GCCC	0.059	0.5 (0.1-2.3)	0.375
12	rs11121278	G/C	1.2 (0.5-3.1)	0.681		GCAC	0.031	1.3 (0.2-8.1)	0.376
13	rs12021597	A/G	1.1 (0.6-2.2)	0.714		GCCT	0.017	1.0 (0.1-11.1)	0.377
14	rs17032912	C/G	1.3 (0.5-3.0)	0.579					
15	rs7545200	G/A	1.3 (0.7-2.6)	0.395	Block 3	GTA	0.361	0.7 (0.4-1.3)	0.238
16	rs1832262	C/T	1.5 (0.8-2.9)	0.237		GCA	0.283	2.1 (1.1-4.1)	0.030
17	rs6691526	A/G	1.1 (0.5-2.2)	0.854		ACG	0.272	0.9 (0.5-1.9)	0.854
18	rs3765965	T/C	2.0 (0.9-4.8)	0.102		ACA	0.083	0.5 (0.1-1.7)	0.241
19	rs2274334	T/G	1.7 (0.5-5.8)	0.394					
20	rs10864376	T/C	2.0 (1.1-3.9)	0.031	Block 4	CCC	0.400	0.7 (0.4-1.3)	0.243
21	rs3737665	T/C	2.0 (1.0-4.1)	0.051		CCG	0.261	0.7 (0.3-1.4)	0.307
22	rs12138897	G/C	1.5 (0.8-2.8)	0.243		TTG	0.228	2.0 (1.0-4.1)	0.051
23	rs3765964	A/G	1.4 (0.8-2.6)	0.285		TCG	0.111	1.4 (0.5-3.5)	0.528
24	rs6577546	G/A	1.0 (0.5-2.0)	0.810	Block 5	AG	0.431	0.9 (0.5-1.7)	0.764
25	rs6680186	A/G	1.0 (0.6-1.9)	0.926		AA	0.309	1.1 (0.5-2.1)	0.889
26	rs7533137	A/G	1.1 (0.5-2.4)	0.757		GA	0.252	1.0 (05-2.0)	0.966
27	rs7513804	T/C	1.2 (0.6-2.3)	0.605					

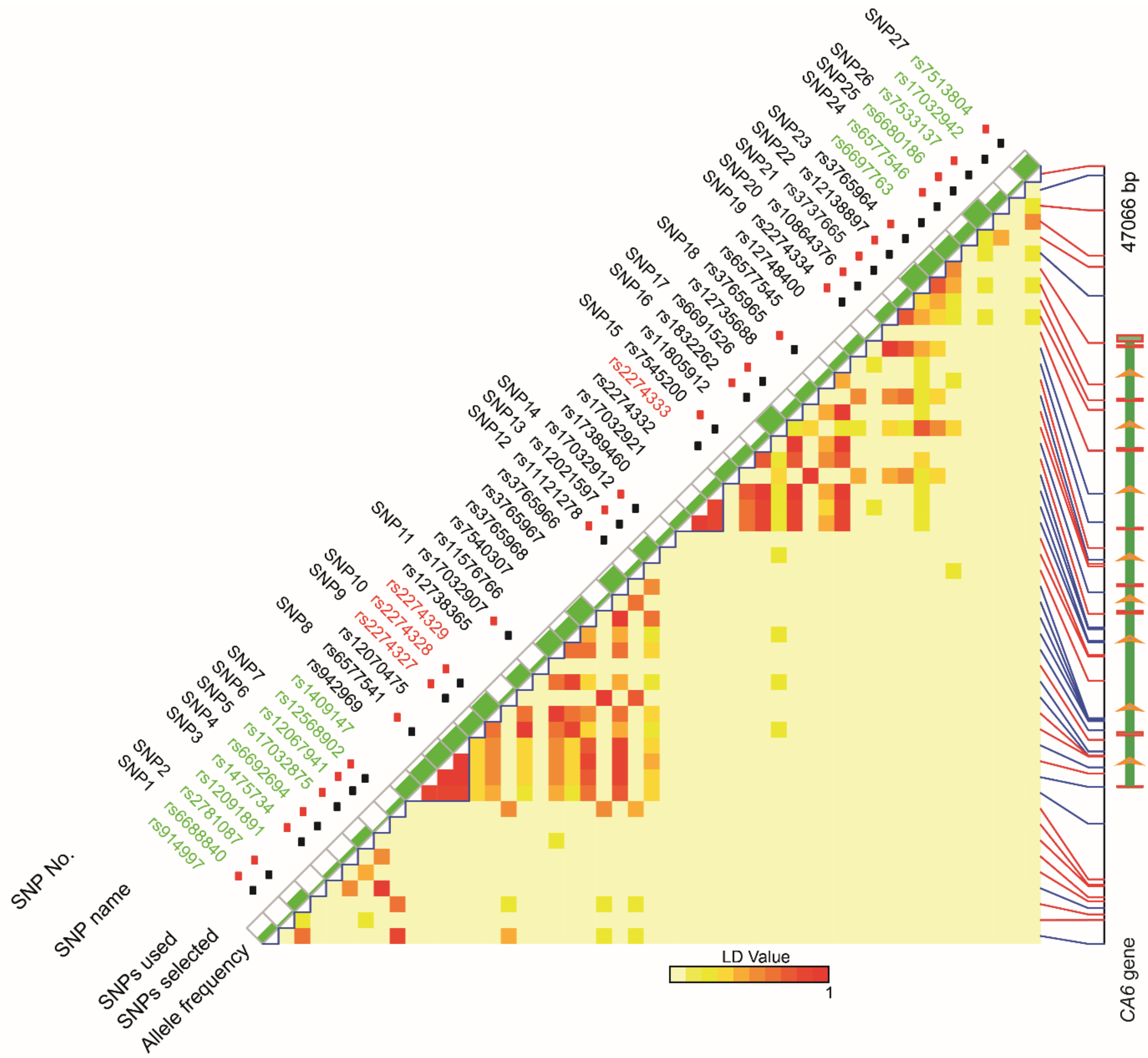

Figure S1. ***CA6*** **single nucleotide polymorphisms (SNP) used in this study.** The *following criteria* for SNP *selection* were employed; minor allele frequency of 0.05 in the CEU population, Linkage disequilibrium (LD) threshold of = 0.8, Minimum 5 of valid genotyping pairs required to calculate LD, Minimum 5 of valid genotyping pairs required to calculate LD, Maximum distance (bp) between SNPs for calculation of LD: 250.000, 10,000 bp up- and down-stream of the *CA6* gene using the NIH snptag program (https://snpinfo.niehs.nih.gov/snpinfo/snptag.html). SNPs name colour indicates, green: up- and downstream of the *CA6* gene; black and red: within gene; red: amino acid change. SNPs selected (n=30). SNPs used (n=27): three SNPs were not included based on call rate 0% (rs2274333 and rs17032942) and deviation from Hardy-Weinberg equilibrium (rs6697763).
